# Efficacy and safety assessment of orthopedic device (LSM-01) for low back pain: A randomized, single-blinded, sham-controlled, parallel-group, pilot clinical trial

**DOI:** 10.1097/MD.0000000000031068

**Published:** 2022-10-28

**Authors:** Chae Hyun Park, Jae Hui Kang

**Affiliations:** a Department of Acupuncture and Moxibustion Medicine, College of Korean Medicine Daejeon University, Daehak-ro, Dong-gu, Daejeon, Republic of Korea; b Department of Acupuncture and Moxibustion Medicine, Cheonan Korean Medicine Hospital of Daejeon University, Notaesan-ro, Seobuk-gu, Cheonan-si Chungcheongnam-do, Republic of Korea.

**Keywords:** low back pain, LSM-01, randomized controlled trial, stiffness

## Abstract

Low back pain refers to pain that occurs mainly in lumbosacral spine, and is a clinically common symptom that 70% to 90% of people experience at least once during their lives.

**Objectives:**

We investigated the effect and safety of orthopedic device (LSM-01) for alleviate back pain caused by muscle tension in a randomized, single-blinded, sham-controlled, parallel-group, pilot trial to establish a basis for large-scale clinical trial.

**Methods:**

A total of 30 subjects were randomly assigned to 2 group: treatment group (LSM-01) and control group (sham device) received treatment with each device 2 to 3 times a week for a total of 6 treatment for 2 weeks. Primary outcome was visual analog scale (VAS); Secondary outcomes were pressure pain threshold (PPT), oswestry disability index (ODI), and patient global impression of change (PGIC). Statistical analysis is performed for full analysis set (FAS) population. Analysis of covariance (ANCOVA) for mean difference of VAS change and Mixed models of repeated measurements (MMRM) for the trend of VAS change were conducted to compare the differences between 2 groups before and after participants got treatment with the clinical trial device.

**Results:**

One participant dropped out due to personal reason and 29 participants completed the clinical pilot trial. We found that the degree of low back pain (VAS, PPT, PGIC) significantly decreased and after the trial in the treatment group compared to the control group. Also, there were no any side effects.

**Conclusion:**

LSM-01 can be effective in improving pain of low back pain. A future large-scale main trial will be conducted based on this pilot study results.

## 1. Introduction

Low back pain (LBP) is a clinical condition related with pathologic pain excluding pain caused by pregnancy or infection in the spinal column below the 10th thoracic vertebrae.^[1]^ More than 80% of the population suffer from LBP at least once during their lives, and 10% to 40% of patients with LBP progress to chronic LBP (lasting >3 months even after healing of primary damaged tissues).^[2]^ It can be caused by numerous factors such as severe exercise, spinal disease, aging, wrong posture, weakened muscles and lesions of internal organs, however most cases of LBP are caused by musculoskeletal abnormalities.^[3]^

In Korean medicine, many acupuncture points exist that strengthen the musculoskeletal system in the low back for the treatment of LBP, the most commonly used are derived from the meridians including bladder meridian (BL), governor vessel (GV), and gall bladder meridian (GB).^[4]^ These meridians pass through lower back muscles such as the erector spinae muscle, gluteus medius, tensor fasciae latae muscle and spinous process. The knowledge of these muscles and treatment points are used in acupuncture and Chuna manual therapy to relieve muscle stiffness and strengthen the muscles.^[5,6]^

An orthopedic device (LSM-01) is a rotating roller machine that uses electricity to mechanically stimulate the muscles and fascia to relieve stiffness and eventually improve pain around the back, spine, pelvis and hip joints. The acupuncture points around the lower back to treat pain include GV3, GV4, GV6, GV9, GV12, GV14, GB30, GB31, GB32, GB33, BL13, BL17, BL20, BL23, BL25, BL26, BL42, BL46, BL49, and BL52. This study aimed to verify the efficacy of LSM-01 in improving LBP by relieving muscle tension, indirectly caused by various causes. This clinical trial was a pilot trial and the preparatory stage for the main clinical trial. The results of this clinical trial provide basic data for future large-scale clinical trials.

## 2. Material and Methods

### 2.1. Study design

This study was a randomized, single-blinded, sham controlled, parallel-group clinical trial (registration number: CRIS-KCT0006425) that examined the efficacy and safety of LSM-01 in improving LBP. This pilot clinical trial was conducted at Cheonan Korean Medicine Hospital of Daejeon University. The clinical trial protocol was designed in compliance with the Standard Protocol Items: Recommendations for Interventional Trials (SPIRIT) 2013 checklist. The study protocol was approved by the Institutional Review Board (IRB) of Daejeon University Cheonan Korean Medicine Hospital (DJUMC-2021-MD-01-1) and has been published.^[7]^ The protocol version 1.1 was approved by the Institutional Review Board (IRB) on July 1, 2021, patient recruitment began in July 2021, and the trial ended in September 2021.

### 2.2. Objective and sample calculation

To perform large-scale trials, prior evidence of basic information for safety and sample count calculations must be obtained. Hence, pilot clinical trial was designed to confirm the efficacy and safety of LSM-01 on improving LBP caused by muscle tension. Thirty patients with LBP were recruited from Daejeon University Cheonan Korean Medicine Hospital through banner promotion. All participants received a complete written explanation of the study protocol and informed consent was obtained by the investigator.

Based on preliminary and parallel-design studies, Julious^[8]^ suggested the empirically optimal sample size of the pilot trial as 12 individuals per group, considering feasibility, estimated average, and dispersion accuracy. When a dropout rate of 20% was considered, the calculated sample size for this pilot clinical trial came to 15 participants per group. Hence the pilot study was conducted after recruiting 30 participants (15 per group) who met the selection criteria.

### 2.3. Inclusion and exclusion criteria

The inclusion criteria were as follows: age: 20 to 70 years; visual analog scale (VAS) ≥40 mm and <75 mm; those who had taken painkillers for LBP; applicants who provided written consent to be a participants of this study.

The exclusion criteria were as follows: history of spinal fractures; plan or history of spinal surgery; severe back pain with VAS ≥75 mm lasting for >3 days within 3 months; neurological symptoms of sensory and motor paralysis; abnormalities detected on lumbar spine (L-spine) computed tomography (CT) and X-ray examination; severe pain in parts of the body other than the low back; skin diseases, inflammations or wounds around the spine, and hip joint; other treatment for LBP except painkillers; severe cardiovascular disease, tumors, alcoholism, and drug abuse; inability to fill out questionnaires by themselves or under the supervision of a guardian/researcher; participants of other clinical trials ≤30 days prior to screening.

### 2.4. Randomization and masking

The participants who met the criteria were assigned to either of the two group — the LSM-01 or sham device group, using SAS statistical software version 9.4 (SAS Institute. Inc., Cary, NC) with balanced block randomization without stratification. Randomized identification codes (e.g., LSM-*R*-001, LSM-*R*-002……, LSM-*R*-030) were assigned to the participants in the order of their recruitment. The trials was conducted by investigators who had not performed the interventional procedures and randomization, they were also separated from the operator and evaluator of the clinical trial. Information regarding the assignment of interventions was stored in an independent secure place. The randomization code was placed in an opaque envelope. Moreover, the trial investigator never served as an operator for this study.

### 2.5. Device

**Figure 1.**
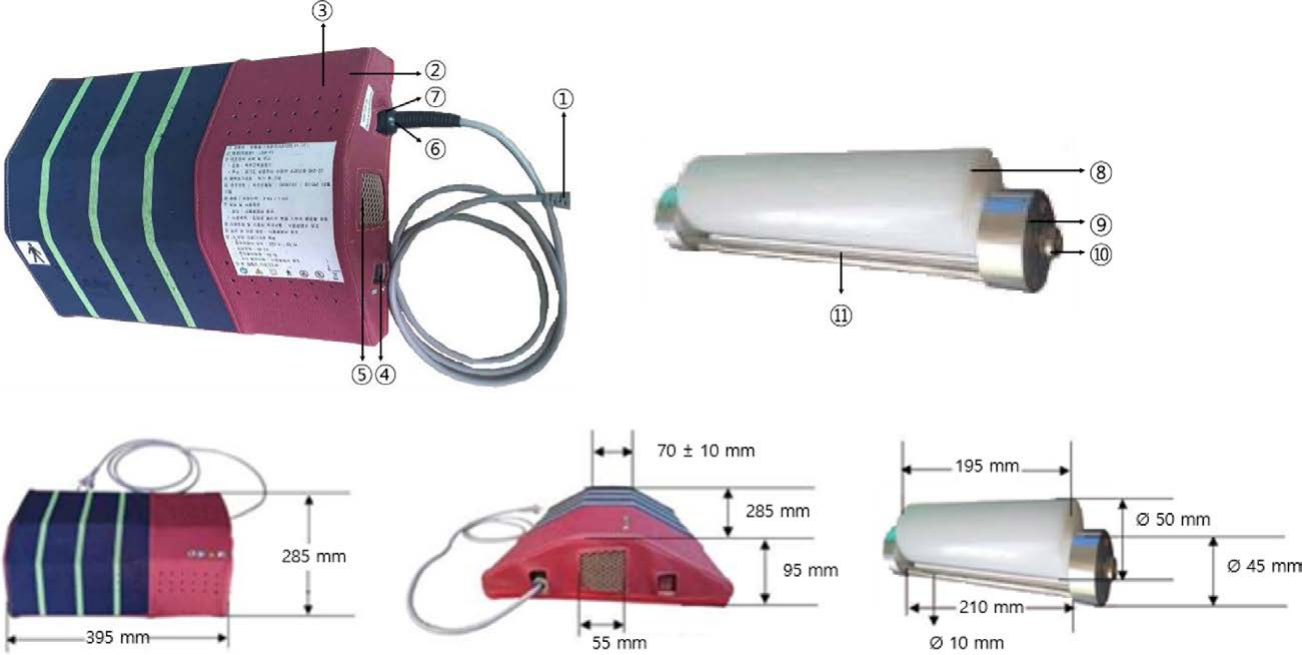
Appearance and size of the device.

**Figure 2.**
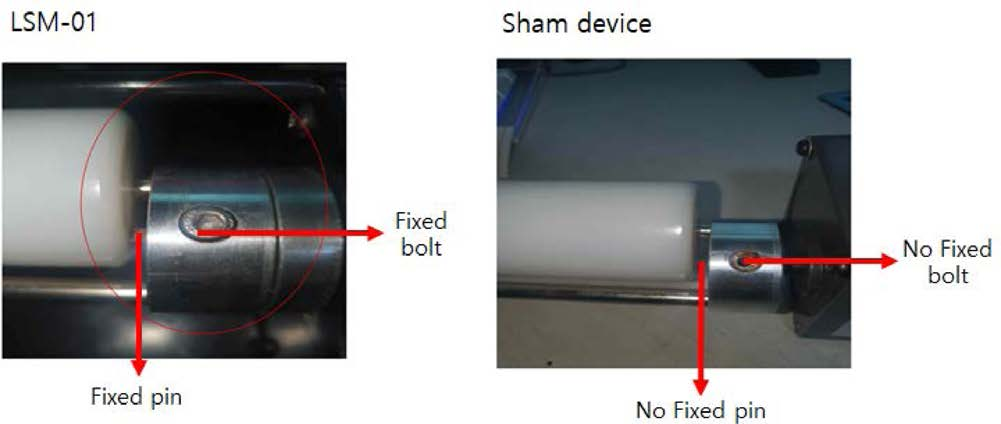
Differences between the LSM-01 and sham device: no fixed bolt and pin.

The device used in this trial was an electrically operated device consisting of a roller, deceleration motor, and a power switch to relieve pain by applying physical force to the affected muscle region (Fig. 1, Table 1). This second-class medical device was licensed by the Ministry of Food and Drug Safety of the Republic of Korea for relieving lumbar muscle pain (classification number: A67025.01). There was no difference in appearance, vibration, or sound between the LSM-01 and the sham device, but the devices differed in the rotation of their rollers because of a variation in connection between the roller and the motor unit (Fig. 2). LSM-01 comprised a functioning roller; however, the sham device lacked fixation between the roller pin and motor, which hindered its rotation. The perception of both the LSM-01 and sham device was similar to ensure blinding, and both devices were manufactured by the Spine Muscle Strengthening Machine Co. (Namyang-ju, Republic of Korea).

**Table 1:** Appearance and description of the device.

①	Power cord	Connects power to the main board
②	Body cover	For safety, a cover protecting the user’s contact part
③	Exhaust vent	Ventilates the interior of the device
④	Power switch	Turns on/off the device
⑤	Body cover	For safety, a cover protecting the user’s contact part
⑥	Cable holder	Fixes the power cable
⑦	Current fuse	Protects the product from excessive current
⑧	Roller	Acts mechanically by direct contact with the human body
⑨	Rotating core	Rotates the roller continuously
⑩	Bearing	Rotates the roller more softly
⑪	Support	Separates roller contact with the human body in each rotation

### 2.6. Intervention

First participants were instructed to lie down in the supine, lateral position at an angle of 45° and LSM-01 was applied 30 times (60 seconds/application) to both gluteus medius muscles alternately. Secondly, in prone, lateral position at an angle of 45°, the LSM-01 was applied 15 times (30 seconds/application) to both tensor fasciae latae muscles alternately. Finally, the LSM-01 was applied, with participants in the supine position, 15 times (30 seconds/application) to the spinous processes of S1, L5, L3, L1, T11, T8, T4 and C7. Same process was followed with the sham device. The intervention was repeated twice per session. A single qualified Korean medical doctor performed the procedure and all participants received treatment according to their assigned group (i.e., LSM-01 or sham device group). The treatment was performed 2 to 3 times a week for a total of 6 times. During the trial, only the use of painkillers was allowed, and other treatments which are used to improve LBP, such as acupuncture, moxibustion, herbal medicine and physiotherapy were prohibited. Concomitant drugs that were not expected to affect the results of this clinical trial (including therapeutic drugs for other diseases or adverse events) were permitted at the discretion of the investigator. If a participant consumed a drug without prior intimation to the investigator, and if the investigator considered that the drug could have a significant impact on the results, that participant was dropped out.

### 2.7. Protocol and outcomes

During screening, a medical history of LBP and other ailments within the last 3 months and drugs consumed within the last 4 weeks were recorded, X-ray and CT imaging of the L-spine was performed for those with LBP based on physical examination, neurological examination, or history. Vital signs and outcome measures were assessed at each visit. Of those who met the enrollment criteria, 30 patients with LBP were randomly assigned in a 1:1 ratio to the treatment group (LSM-01) and the control group (sham device). The interaction started within 2 weeks of the screening visit. The participants were treated with either LSM-01 or sham device for 2 to 3 weeks. The total planned duration of the trial was 3 to 4 weeks. The subjects were assessed for efficacy and safety at every visit. The primary outcome measures was VAS score, which was used to evaluate severity of LBP. Secondary outcome measures included the average change in the pressure pain threshold (PPT), Oswestry disability index (ODI) after final treatment (V7) compared with baseline (V1), and patient global impression of change (PGIC) after treatment compared with just before treatment. VAS, PPT, ODI, and PGIC scores were measured at every visit. VAS is mainly used to subjectively evaluate the intensity and frequency of pain. It is used by the patient himself, to indicate pain severity on a 10-cm horizontal straight line, higher score indicates more severe pain. PPT is an index to measure the pressure (kg/second/cm^2^) at the moment when a patient first complains of pain when pressed vertically on both sides of the back at a rate of 1 kg/second, with a pressure algometer. A higher score indicates that the patient has an increased threshold for pain. ODI indicates dysfunction due to LBP; it consists of 10 contents with six questions, each question with a score ranging from 0 to 5, and the subject marks the relevant content. A higher score indicates a more severe disability. The PGIC is a questionnaire for assessing the improvement by the patient himself, before and after the treatment. There is a flow chart (Table 2).

**Table 2:** Flow chart of study.

	Study period				
	Recruitment	Intervention period			Observation period
Visit	Screening	V1 Before intervention	V1 After intervention	V2-V6	V7
Checking the selection/exclusion criteria	●				
Vital signs	●	●		●	●
Demographic surveys and body measurements	●				
Medical history of lumbar and other body organs	●				
Lumbar spine X-ray and computed tomography (CT)	●				
Physical examination	●				
Random assignment		●			
Treatment (Orthopedic or sham device)		●		●	

### 2.8. Data analysis

All statistical analyses were performed for the full analysis set (FAS), and the per protocol (PP) set group analysis was performed as the supplementary analysis. Analysis of covariance (ANCOVA) using the baseline value of VAS as a covariate was performed to assess the average change in VAS scores from baseline (V1) to the end of 30 days (V7) with the LSM-01 and sham device groups. The average mean, standard error, 95% confidence interval, t statistic, and *P* value were determined for each group. A linear mixed-effects model for repeated measures (MMRM) was used to ascertain the trend of the VAS score changes, the secondary outcome measures were analyzed in the same manner. The analysis was evaluated with a 95% confidence interval, and the significance level of statistical analysis was α = 0.05. The missing data was handled by using last observation carried forward analysis (LOCF) to analyze the outcomes. All statistical analyses were performed using SPSS Statistics for Windows Version 20.0 (IBM Corp., Armonk, NY).

Safety evaluation was mainly performed by analyzing the incidence of treatment-related occurrence of adverse events (AEs) and serious adverse events (SAEs) suspected by the investigator. An independent sample *t* test or Wilcoxon’s rank-sum test was performed to analyze continuous variables; the McNemar test was used to analyze categorical variables. The chi-squared test or Fisher’s exact test was performed to examine the potential association of the treatment with the occurrence of AEs and SAEs.

### 2.9. Withdrawal and dropout

Participants who violated the inclusion criteria during the trial; withdrew their consent; took prohibited medicines and treatments; or became pregnant were excluded from the study. A participant would drop out if SAEs occurred or it was difficult to continue the clinical trial due to AEs. In all cases of dropouts, causes were recorded in detail in the case report forms. Incidence of an AE was followed-up until the cause of the abnormal reaction was identified, and the results recorded. If a SAE occurred and the participant dropped out, the IRB was notified immediately.

## 3. Results

**Figure 3.**
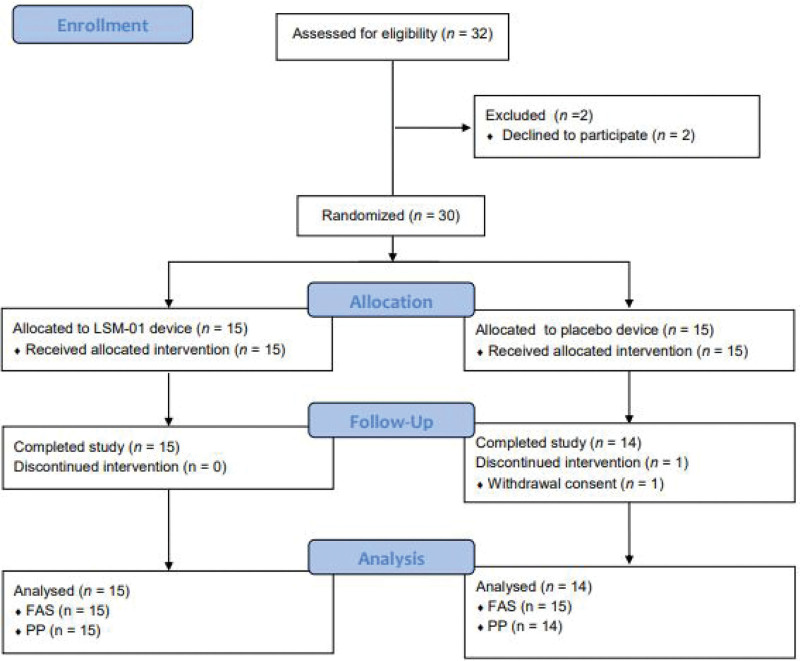
CONSORT 2010 flow diagram.

From August 11, 2021 to September 23, 2021, 32 individuals volunteered to participate in this clinical trial, 30 of whom were recruited and randomly assigned to two groups—the LSM-01 (n* *= 15) and sham device (n = 15) groups. One participant from the sham device group dropped out for personal reasons; consequently, 29 participants completed the trial (Fig. 3). The PP group comprised 29 participants (LSM-01: 15, sham device: 14) and the FAS group comprised 30 participants (LSM-01: 15, sham: 15). There were no significant differences in the demographic and clinical characteristics of the participants in each group (Table 3).

**Table 3:** Subject characteristics in baseline.

Characteristic	Control, N = 151	Treatment, N = 151	*p* value^2^
Sex: Female, n (%)	12 (80%)	13 (87%)	>.999
Age, Mean (SD)	49 (12)	40 (9)	.024
Weight (kg), Mean (SD)	65 (11)	61 (12)	.263
Height (cm), Mean (SD)	163 (10)	162 (6)	>.999
BMI (kg/m²), Mean (SD)	24.3 (4.0)	23.0 (3.0)	.481
Systolic BP (mm Hg), Mean (SD)	118 (12)	116 (8)	.724
Diastolic BP (mm Hg), Mean (SD)	72 (7)	71 (7)	.983
Pulse rate (bpm), Mean (SD)	79 (9)	81 (10)	.417
Body Temperature(°C), Mean (SD)	36.57 (0.31)	36.68 (0.28)	.276
VAS at Screen (mm), Mean (SD)	49 (8)	50 (9)	.663
SLRT: Left, Mean (SD)	82.0 (4.1)	85.3 (5.2)	.066
SLRT: Right, Mean (SD)	82.0 (4.1)	85.3 (5.2)	.066
Study Duration (days), Mean (SD)	19.47 (5.03)	21.33 (1.54)	.259
Previous treatment for LBP, n (%)			
Tablet	3 (20%)	1 (6.7%)	.598
Physiotherapy	11 (73%)	8 (53%)	.256
Chiropractic	5 (33%)	2 (13%)	.390
Korea Medicine	13 (87%)	11 (73%)	.651
Previous Korean Medicine treatment for LBP, n (%)			
Acupuncture	13 (100%)	11 (100%)	>.999
Cupping	9 (69%)	9 (82%)	.649
Herbal medicine	3 (23%)	2 (18%)	>.999
Moxibustion	3 (23%)	7 (64%)	.095

LBP = low back pain, VAS = visual analog scale.

**Figure 4.**
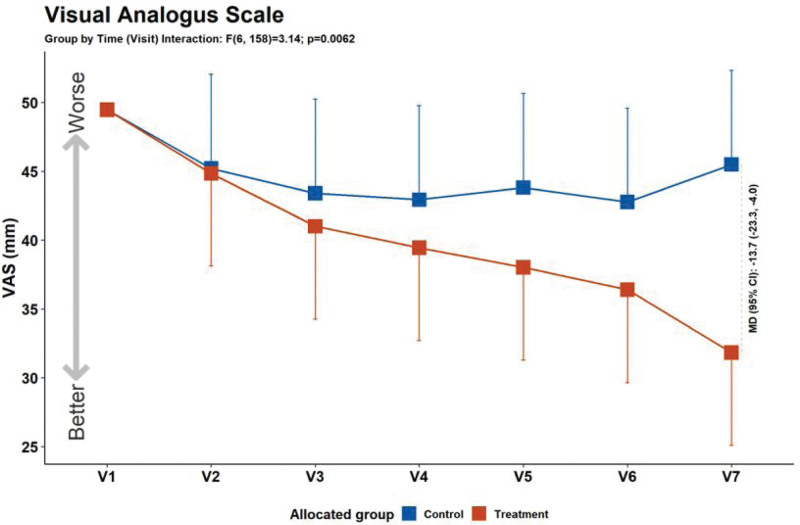
Primary outcome: VAS score in both groups. VAS = visual analog scale.

**Figure 5.**
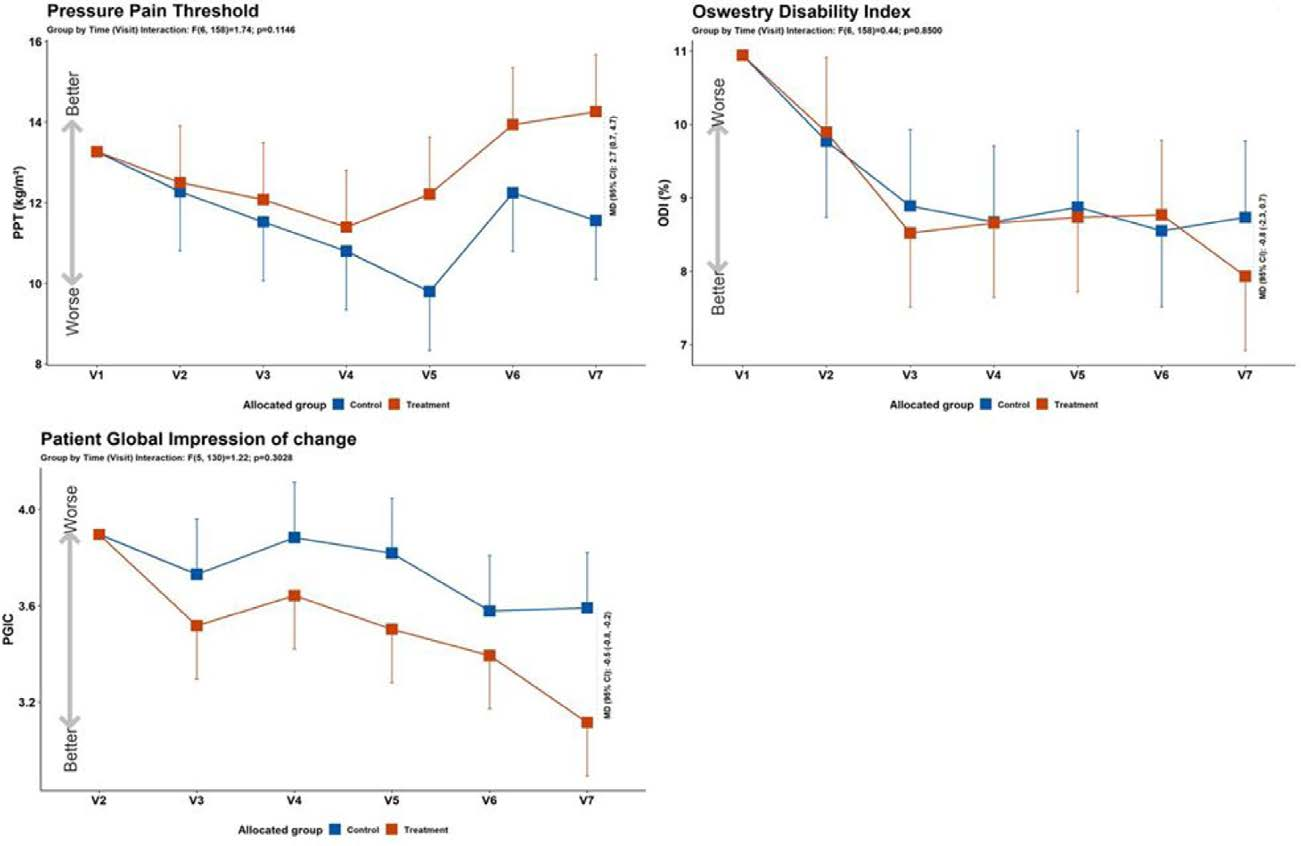
Secondary outcomes: PPT, ODI, PGIC. ODI = oswestry disability index, PGIC = patient global impression of change, PPT = pressure pain threshold.

The primary outcome, the VAS score, was compared at baseline (V1) and after 30 days of treatment (V7), and decreased significantly in the LSM-01 group (Fig. 4, Table 4). Regarding secondary outcomes, the PPT (V1 vs V7) and PGIC (V2 vs V7) scores showed statistically significant differences. The change in the ODI scores in LSM-01 group was significant, but there was no statistically significant difference between the LSM-01 and sham device groups (Fig. 5, Table 4).

**Table 4:** Outcomes comparison between and within each group (FAS group).

Variable		Observed value		Change from baseline		
		Control, N = 15	Treatment, N = 15	Control, N = 15	Treatment, N = 15	*p* value†
**VAS**	Visit 1	49.2 ± 7.8	50.3 ± 8.5			
	Visit 7	46.1 ± 15.9	32.1 ± 13.4	3.4 ± 11.1	17.9 ± 25.6	.011
	***p* value** *	.374	**<.0001**			
**PPT**	Visit 1	13.5 ± 5.0	13.4 ± 3.2			
	Visit 7	12.3 ± 4.7	14.3 ± 3.0	-1.4 ± 2.8	0.9 ± 0.6	.029
	***p* value** *	-.053	.226			
**ODI**	Visit 1	10.2 ± 3.5	11.8 ± 2.5			
	Visit 7	8.9 ± 3.9	8.7 ± 3.2	2.0 ± 3.3	3.0 ± 4.3	.259
	***p* value** *	.002	**<.0001**			
**PGIC**	Visit 2	4.0 ± 0.4	3.8 ± 0.6			
	Visit 7	3.6 ± 0.6	3.1 ± 0.6	0.3 ± 0.6	0.8 ± 1.1	.038
	***p* value** *	.059	**<.0001**			

ODI = oswestry disability index, PGIC = patient global impression of change, PPT = pressure pain threshold, VAS = visual analog scale.

* *P* values were compared between V1 and V7 in each group (independent *t* test).

† *P* values were compared between groups (independent *t* test).

Two AEs were reported: gastroesophageal reflux disease and rib sprain. All AEs were resolved with no consequent dropouts and retained blinding. No association between the AEs and the LSM-01 was found; additional safety evaluation through physical examination, indicated that LSM-01 was not a clinically harmful device.

## 4. Discussion

LBP refers to pain that occur mainly in the lumbar vertebrae. Compared with other vertebrae, the L-spine receives the most tension and pressure associated with supporting weight.^[9]^ Recently as the prevalence of sedentary lifestyle has increased due to the increase in computer usage and decrease in the magnitude of exercise, and maintaining one posture for a long time can induce excessive tension in the lower back.^[10]^ More than 80% of people worldwide experience LBP at least once in their lifetime, and the annual average prevalence is reported as 30%.^[11,12]^ An unstable posture disturbs the balance of the human body as compensation for gravity, displacement occurs and the musculoskeletal system receives more load on one side.^[13]^ It is reported that the load on one side creates excessive tension in the muscles, which stiffens the muscle fibers, delays blood and lymph circulation, damages tissues, and interferes with most functions of daily living that work against gravity.^[14,15]^

In Korean medicine, the meridian is a line that connects points to maximize therapeutic effect by activating the flow of energy in the body, and is widely used in remedying various diseases.^[16,17]^ Among the meridians passing through the back, the BL, GV, and GB are frequently used for the treatment of LBP.^[4]^ These meridians coincide with the location of the lower back muscles. The gluteus medius and tensor fasciae latae are muscles that attach to the iliac bone and are involved in the stability of the lower back and pelvis. If these muscles become tense and do not function properly, it can cause LBP.^[18,19]^ In this study, the treatment included the use of acupoints (GV3, GV4, GV6, GV9, GV12, GV14, GB30, GB31, GB32, GB33, BL13, BL17, BL20, BL23, BL25, BL26, BL42, BL46, BL49, and BL52), gluteus medius and tensor fasciae latae.

The orthopedic device (LSM-01) used in this study is a second degree medical device with a rotating roller machine that uses electricity. This rotating roller stimulates the muscles and fascia mechanically and physically to relieve stiffness and eventually improve pain around the back, spine, pelvis, and hip joints. Not only does it relieve muscle tension but it also promotes blood circulation and aids in tissue damage recovery.

To evaluate the effect of LSM-01, VAS, PPT, ODI, and PGIC scores were used to assess subjective pain intensity, frequency, degree of improvement, and pain threshold, which can be expressed as objective values.^[20–23]^ At screening, using CT scans and X-rays, participants with LBP due to causes other than muscle tension, such as herniated disc or stenosis of the spine were excluded. In VAS, the primary outcome measure, both the control and treatment groups showed a decrease in scores (V1 vs V7) and when compared to the control group, the treatment group showed a greater improvement which was statistically significant. Among the secondary outcome measures, PPT and PGIC also showed statistically significant improvement, and the treatment group showed a significantly greater improvement compared to the control group. The ODI score also improved in both the control and treatment groups, however, there were no significant differences between the control and treatment groups. Safety was evaluated based in AEs, and no hazards were observed.

In all outcome measures, it was confirmed that LBP can be improved by stimulating the muscles with LSM-01 to relieve tension. However this study has limitations being a simple before-and-after comparative study, and future comparative studies with a control group with no treatment or with LBP due to causes other than muscle tension are warranted.

## 5. Conclusion

The results obtained after six treatments indicated that LSM-01 was more effective in alleviating LBP induced by muscle tension than the sham device. Although there are many studies on medical devices for treating back pain, there are few studies on medical devices targeting acupuncture points or meridians for LBP treatment. As meridians and acupuncture points are important treatment areas with proven efficacy, this study has significance not only from the perspective of Korean medicine but also from the perspective of alternative and complementary medicine. Therefore, this study can serve to promote the development of Korean medicine-related industries and medical devices that can be easily used without side effects.

## Author contributions

CHP performed the trial, acquired and analysis the data and wrote the article. JHK designed, administered, and supervised the clinical trial, and critically revised the manuscript. Both authors have read and approved the final version of the manuscript.

**Supervision:** Jae Hui Kang.

**Writing – original draft:** Chae Hyun Park.

**Writing – review & editing:** Chae Hyun Park.

## References

[1] Molumphy M Unger B Jensen GM. Incidence of work-related low back pain in physical therapists. Phys Ther. 1985;65:482–6. 3157196 10.1093/ptj/65.4.482

[2] Dugan SA. The role of exercise in the prevention and management of acute low back pain. Clin Occup Environ Med. 2006;5:615vi–32vii. 16963378 10.1016/j.coem.2006.03.003

[3] Goo BO Park MC Song YY. Effect of 8 direction incline and rotation exercise on pain and dynamic balance in the patients with chronic low back pain. Int J Cont. 2010;10:285–92.

[4] Nam DJ Huh G Lee HE. Systematic review of high frequency of acupuncture point and self exercise therapy for lower back pain. J Kor Med Rehab. 2013;23:59–71.

[5] Lee SH Cheong BS Yun HS. Therapeutic effect of Weizhong(BL40) venepucture on low back pain. J Kor Acup Moxibust Soc. 2002;19:65–75.

[6] Lim DC Oh JK Jeon KK. Effects of Chuna therapy and spinal stabilization exercise on muscle areas of lumbar spine and body stability with chronic low back pain patients. Kor J Sports Sci. 2012;21:1215–25.

[7] Won ES Kang JH. Assessment of the efficacy and safety of an orthopedic device for low back pain: study protocol for a randomized, single-blinded, sham-controlled, parallel-group, pilot clinical trial. Medicine. 2022;101:e28527. 35060507 10.1097/MD.0000000000028527 PMC8772674

[8] Julious SA. Sample size of 12 per group rule of thumb for a pilot study. Pharmaceut Stat. 2005;4:287–91.

[9] Song HS Kang MS Park DS. The Acupuncture and moxibustion medicine. Paju. 2012:506–25.

[10] Jensen MC. Magnetic resonance imaging of the lumbar spine in people without back pain. N Eng J Med. 1994;331:69–73. 10.1056/NEJM199407143310201 8208267

[11] Waxman R Tennant A Helliwell PA. Prospective follow-up study of low back pain in the community. Spine. 2000;25:2085–90. 10954640 10.1097/00007632-200008150-00013

[12] Marras WS. Occupational low back disorders causation and control. Ergonomics. 2000;42:880–902. 10.1080/001401300409080 10929824

[13] Shin J.S. Korean Chuna Studies Clinical Standard Guidelines. 3rd ed. Korean: Korean Society of Spinal Neurology. 2019

[14] Yuk JY Lee YJ. Effects of stretching, beauty massage on recoveries of work capacity and blood lactate after strenuous exercise. Korea Sports Res. 2005;6:467–86.

[15] Richardson CA Jull GA. Muscle control-pain control. what exercises would you prescribe. Man Ther. 1995;1:2–10. 11327788 10.1054/math.1995.0243

[16] Yun SJ Pack SH Shin MH. Research paper: the effect of the Meridian massage on the change of the low back pain RPE (Rating of Perceived Exertion) and the changes of serum cortisol as well as stress index of middle aged women. J Kor Soc Cosmetol. 2013;19:751–6.

[17] Jun JY. The effects of Kyongrak massage in the elderly with chronic pain. Kor J Rehabilit Nurs. 2001;4:155–64.

[18] Arokoski MH Arokoski JP Haara M. Hip muscle strength and muscle cross sectional area in men with and without hip osteoarthritis. J Rheumatol. 2002;29:2185–95. 12375331

[19] Dwyer MK Stafford K Mattacola CG. Comparison of gluteus medius muscle activity during functional tasks in individuals with and without osteoarthritis of the hip joint. Clin Biomech. 2013;28:757–61. 10.1016/j.clinbiomech.2013.07.007 23911109

[20] Shin SY Park HJ Lee JM. An overview of pain measurements. Kor J Acup. 2007;24:77–97.

[21] Moon BH Choi YJ Yoo SB. A literatural investigation of diagnosis methods and evaluation outcomes for the clinical trials on temporomandibular disorders. J Kor Med Rehab. 2016;26:45–55.

[22] Kim GM Park SY Yi CH. A rash analysis of the Korean version of Oswestry disability questionnaire according to general characteristics of patients with low back pain. Kor Res Soc Phys Ther. 2011;18:35–42.

[23] Farrar JT Young JP La Moreaux L. Clinical importance of changes in chronic pain intensity measured on an 11-point numerical pain rating scale. Pain. 2001;94:149–58. 11690728 10.1016/S0304-3959(01)00349-9

